# The Intertwining of Transposable Elements and Non-Coding RNAs

**DOI:** 10.3390/ijms140713307

**Published:** 2013-06-26

**Authors:** Michael Hadjiargyrou, Nicholas Delihas

**Affiliations:** 1 Department of Life Sciences, Theobald Science Center, Room 420, New York Institute of Technology, Old Westbury, NY 11568, USA; E-Mail: mhadji@nyit.edu; 2 Department of Molecular Genetics and Microbiology, School of Medicine, Stony Brook University, Stony Brook, NY 11794, USA

**Keywords:** non-coding RNAs, transposable elements, microRNAs, Alu sequences, endogenous retrovirus LTR, epigenetics, disease formation

## Abstract

Growing evidence shows a close association of transposable elements (TE) with non-coding RNAs (ncRNA), and a significant number of small ncRNAs originate from TEs. Further, ncRNAs linked with TE sequences participate in a wide-range of regulatory functions. Alu elements in particular are critical players in gene regulation and molecular pathways. Alu sequences embedded in both long non-coding RNAs (lncRNA) and mRNAs form the basis of targeted mRNA decay via short imperfect base-pairing. Imperfect pairing is prominent in most ncRNA/target RNA interactions and found throughout all biological kingdoms. The piRNA-Piwi complex is multifunctional, but plays a major role in protection against invasion by transposons. This is an RNA-based genetic immune system similar to the one found in prokaryotes, the CRISPR system. Thousands of long intergenic non-coding RNAs (lincRNAs) are associated with endogenous retrovirus LTR transposable elements in human cells. These TEs can provide regulatory signals for lincRNA genes. A surprisingly large number of long circular ncRNAs have been discovered in human fibroblasts. These serve as “sponges” for miRNAs. Alu sequences, encoded in introns that flank exons are proposed to participate in RNA circularization via Alu/Alu base-pairing. Diseases are increasingly found to have a TE/ncRNA etiology. A single point mutation in a SINE/Alu sequence in a human long non-coding RNA leads to brainstem atrophy and death. On the other hand, genomic clusters of repeat sequences as well as lncRNAs function in epigenetic regulation. Some clusters are unstable, which can lead to formation of diseases such as facioscapulohumeral muscular dystrophy. The future may hold more surprises regarding diseases associated with ncRNAs andTEs.

## 1. Introduction

The genome is a dynamic entity, ever-changing as a result of endogenous DNA movement or the acquisition of exogenous DNA leading to genomic rearrangements. Such events have contributed to the plasticity and evolution of the genome and all of its complexity, much of which has slowly come to light over the past decades but whose pace has certainly accelerated in the last few years as a result of breakthroughs in genomic technologies, development of newer sequencing techniques, and availability of data in public databases by scientists all over the world. This new and vast genomic knowledge has led to a revolutionary and unparalleled in depth examination of the genome, transcriptome, proteome, interactome, *etc.* Indeed, the concept and definition of a gene may have to be altered [1,2].

However, one key question before us is what new functional loci and regulatory mechanisms have been formed during genomic evolution, especially as they pertain to the genesis of non-coding RNAs (ncRNAs), ncRNA regulatory roles and their association and interaction with transposable elements (TEs). In this review, we outline recent advances in origins of microRNAs (miRNA) and functional properties of ncRNAs as they pertain to their interaction with TEs, especially in humans. What emerges is a fascinating new picture of interconnected molecular interactions and regulatory pathways.

### 1.1. Non-coding RNAs

The development and use of new sequencing techniques, such as RNA-Seq has greatly increased our discovery of new RNAs [3,4]. The Encyclopedia of DNA Elements (ENCODE), an international project with the intent goal of determining functional elements of the entire human genome, has employed these techniques and found thousands of new RNA transcripts [2,5]. Surprisingly, about 75%–85% of the human genome is transcribed into primary and processed transcripts [2]; yet only 1.2% of the human genome encodes proteins. This suggests that most of the human genome space is devoted to RNA synthesis that is not devoted to protein-coding. Functions for most non-coding RNA (ncRNA) transcripts are unknown, but the future may hold fascinating prospects of finding new roles and molecular pathways. For example, thousands of circular ncRNAs (cirRNA) have recently been identified; they represent scrambled coding sequences that originate from exons (nonrandom products of RNA splicing) and are involved in small ncRNA regulation [6,7]. These cirRNAs are transcripts that do not encode proteins but have a regulatory role in the cell and thus are regulatory ncRNAs. A significant number of ncRNAs stem from non-protein-coding regions of the genome (intergenic regions), but many also originate from protein-coding regions as antisense transcripts, or from intron regions, and as just mentioned, from scrambled coding sequences. Many ncRNAs target mRNAs and induce their degradation. On the other hand, others are associated with regulation of transcription. Indeed the biological significance of regulation by RNA was grossly underestimated in the past.

Given the barrage of published studies on newly discovered ncRNAs, especially with eukaryotes, their classification and subclassification is indeed very challenging. However, Di Leva and Garofalo [8] used a simple classification system and presented three basic categories: (1) Housekeeping RNAs (rRNAs, tRNAs, snRNAs and snoRNAs); (2) short non-coding RNAs that are less than 200 nucleotides that include but are not limited to microRNAs (miRNAs), Piwi-interacting RNAs (piRNAs) and retrotransposon-derived ncRNAs, and (3) long non-coding RNAs (lncRNAs) that are greater than 200 nucleotides. lncRNAs are currently divided into long intergenic ncRNAs (lincRNAs) as they are encoded in intergenic regions, transcripts from introns, long ncRNA that are antisense transcripts in coding regions but do not encode proteins, and circular RNA transcripts from coding regions that have scrambled exon sequences and also do not encode proteins. Recent identification and classification of long ncRNAs lists additional categories [5].

A common theme that prevails in target RNA regulation by ncRNAs is the formation of short intermolecular RNA/RNA base-paired stems that contain Watson–Crick pairs, imperfect pairing (bulged and looped out positions), and non-canonical pairs. Imperfect ncRNA/target RNA pairing was determined experimentally with the first discovered and functionally characterized non-coding RNA in prokaryotes [9–11]. This is a type of ncRNA/target RNA binding that prevails throughout all biological kingdoms, although RNA binding proteins are also key factors in stable binding. Most of the ncRNAs discussed in this paper interact with their target RNAs via imperfect pairing.

### 1.2. Transposable Elements

TEs are defined as mobile genetic elements (pieces of DNA capable of moving to new locations); they also constitute the “mobilome” in that they can impact cell transcription [12]. TEs are characterized as either Class I retrotransposons or Class II DNA transposons. Retrotransposons are further subdivided based on the presence of their long terminal repeat (LTR) that contains the element’s functions for mobility and regulatory sequences. LTRs flank endogenous retroviruses (ERV) and are capable of transposition. ERVs are mostly inactive viruses due to accumulation of mutations, but LTRs are active transposons, encode for all the essential factors for mobility and can multiply within a cell independent of the ERV. They carry promoter and enhancer sequences enabling host genes to be transcribed, as well as lncRNA genomic sequences. There are about 500,000 copies of LTR sequences in Homo sapiens, which make up about 8 percent of the human genome.

The other type of Class I retrotransposons are composed of the Long Interspersed Nuclear Elements (LINEs) as well as the Short Interspersed Nuclear Elements (SINEs). LINEs are mobile, whereas SINEs are non-autonomous DNA transposable elements and require LINEs for their mobility and propagation [13]. Duplicate copies are generated during mobility, with sequences identical to the original element inserted in a new location on the genome. In time, such copies may accumulate mutations independently and therefore will differ in sequence from their original sequence leading to increased divergence.

**Figure 1:**
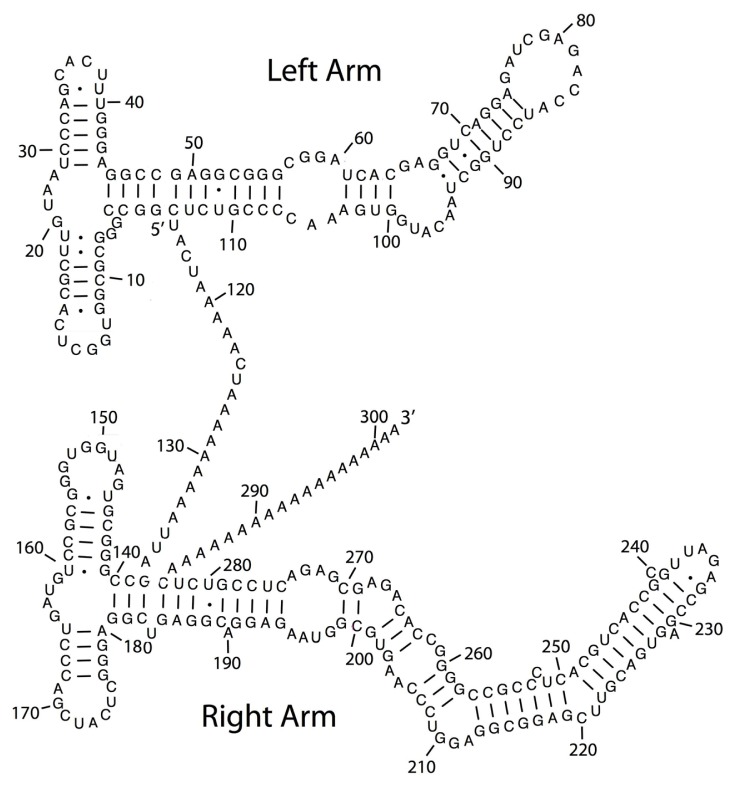
Secondary structural model of Alu RNA. Modified from [15] with permission from Dr. Jennifer Doudna.

SINEs do not encode proteins. Alu sequences are classified as SINE elements and are about 300 nucleotides. They originated from the 7SL RNA transcript via retrotranscription into DNA. 7SL RNA forms part of the signal recognition particle. A hallmark of Alu sequences is that Alu RNAs fold into specific stable stem-loop structures, albeit with extensive imperfect base-pairing (Figure 1). Alus are highly abundant in mammalian cells, e.g., there ~10^6^ copies of Alu in the human genome that make up ~11 percent of the genome [14], but most of these cannot be mobilized due to accumulation of mutations. Alu sequences are also found embedded in lncRNAs, where they are found to directly participate in base-pairing to target mRNAs (see Section 3.1). Additionally they are also found in piRNA genetic clusters (see Section 3.9).

All-in-all, repeat sequences comprise 50%–75% of the human genome [14,16]. Repeats are broadly classified either as TE repeats or tandem repeats [17], but the major fraction of repeats represent transposable elements, either active or inactive. Tandem repeats represent a rather heterogeneous group, but some may have originated from TEs [18]. Repeats are regions where there is high recombination and this may sometimes result in genetic abnormalities.

In this review we focus on the origins of ncRNAs, the microRNAs from TEs and the interaction of ncRNA with TEs, primarily as found in mammalian tissues. Evidence is rapidly accumulating to show that this intimate association plays a central role in molecular and genetic mechanisms, such as RNA-based immunity. Furthermore, Cowley and Oakley have already described some of the impact of TEs in the promotion of human transcript diversity [12].

## 2. TE Origins of miRNAs

microRNAs (miRNA) are small non-coding regulatory RNAs molecules that function post-transcriptionally by binding to the 3′UTR of target mRNAs and ultimately inducing inhibition of target mRNA function. While the majority of miRNAs originate from intergenic genomic sequences, some arise from genes and TEs. The molecular origins of many miRNAs support the hypothesis that miRNA hairpin generation is based on the insertion of two related TEs flanking a single genomic locus (see below). As such, transcription that occurs across this locus leads to the biogenesis of functional miRNAs. One of the earliest studies to indicate that a number of mammalian miRNAs are derived from TEs utilized a bioinformatics approach, where the authors analyzed the Sanger miRNAi database using a software program that specifically detects well characterized repeats [19]. Specifically, 11 different miRNA precursors contained repeat sequences (4 derived from LINE-2 repeats and others with SINEs, LTRs and simple repeats). The majority of these miRNAs are highly conserved across human, mouse and rat, but some are confined to only one or two species.

In a subsequent and more in depth study, Piriyapongsa and Jordan investigated the relationship between human miRNAs and TEs by comparing the genomic locations of experimentally characterized human miRNA genes with the locations of annotated genomic TE sequences [20]. A correlation was observed, and nt sequence comparisons showed a high identity between seven members of the family of miRNAs hsa-mir-548 and the miniature inverted repeat transposable element (MITE), Made1. By use of human genome tilling arrays that visualize genomic expression, one Made1 element was found to be inserted into a transcriptionally active intergenic site. Made1 and other MITEs have palindromic sequences, and when transcribed, show a segment that has an imperfect stem-loop RNA structure. As RNAi-related enzymes can recognize this type of imperfect stem-loop and process it into the 22 bp mature miRNA sequences, the authors proposed that Made1 TE transcripts are processed into hsa-mir-548 miRNAs. The expression date and high sequence identities strongly support the proposed TE origin of several hsa-mir-548 family members.

In a related study, Piriyapongsa *et al*. used comparative genomic sequence data from the UCSC Genome Browser and evaluated the evolution of TE-derived human miRNAs [21]. They found 55 experimentally characterized human miRNA genes that were derived from TEs (LINE and SINE, LTRs and DNA transposons). Sequence comparisons showed that on average, TE-derived miRNAs are less conserved than non-TE-derived miRNAs. Further, a subset of these, are related to the ancient L2 and MIR families. Results also predicted an additional 85 novel TE-derived miRNA genes. Lastly, for some of the TE-derived miRNAs and their putative target genes, a comparison of expression patterns (miRNA *vs.* mRNA) was performed and revealed a number of them to have anti-correlated expression, consistent with regulation via mRNA degradation and thus supporting their regulatory function.

Examination of fourteen previously identified marsupial (Monodelphis domestica) specific miRNAs and their flanking sequences revealed that half of these miRNAs evolved from marsupial-specific TEs [22]. More specifically, six of these TE sequences were identified as LINEs and one as a Mariner DNA transposon. In a subsequent study, Yuan and colleagues also investigated another placental-specific miRNA gene family (miR-1302) that at the time of the analysis had 11 members that were distributed in the human genome (present in the miRBase) [23]. They demonstrated that all members of this family were derived from a single transposon (MER53 element). MER53 is a TE with a 193-bp consensus sequence and is characterized by the presence of terminal inverted repeats and TA target site duplications that can form palindromic structures [24]. Further analysis of the phylogenetic distribution and evolution dynamics of the miR-1302 family identified 36 potential paralogs of MER53-derived miR-1302 genes in the human genome and another 58 potential orthologs in placental mammals and showed that these members of the hsa-mir-1302 family emerged within the last 180 million years since placental mammals diverged from marsupials. Lastly, the authors also explored the targets of the mature human miR-1302 and found 1835 genes with predicted function in transportation, localization, system development processes and their regulation, as well as in binding and in transcription regulation [23].

Genome-wide studies were performed using a comparative genomics approach in order to identify human miRNA paralogs (in mouse and rhesus) in segmental duplication pair data [25]. Of ~1000 miRNA genes and ~1000 mature sequences from human, ~700 miRNA genes and ~1000 mature sequences from mouse, and ~500 miRNA genes and ~500 mature sequences from rhesus, they identified 228 novel miRNA homologs in the rhesus genome and 22 novel miRNA homologs in the mouse genome (by using miRBase 16). Further, they also found 12 and 2 novel miRNA paralogs in the human and mouse genome, respectively, but none were found in the rhesus genome. In a separate analysis, the authors also examined the coverage density of repetitive elements, and if it was at least 50% in a miRNA gene or 100% in one of the associated mature miRNA sequences, then the miRNA gene was considered to be a RdmiR. Using this rule, the study identified a large number of miRNAs genes that overlap with repeats (TEs: LINEs, SINEs and LTRs) and other types of repetitive elements (DNA transposons, specifically MADE1 elements) within the three genomes; 226 (human), 115 (rhesus) and 141 (mouse). The study also identified a smaller number of possible repeat derived miRNAs, which they termed RrmiRs. Lastly, a computational analysis was conducted to investigate the functions of 19 of the conserved RrmiR families (between the three genomes), by identifying their target genes and it was found that the most significant targets are involved in transcriptional regulation, central nervous system development, and negative regulation of biological process. Collectively, the results of this study suggest that repetitive elements contribute to the de novo origin of miRNAs, and that large segmentation duplication events most likely accelerate the expansion of miRNA families (including RdmiRs).

**Figure 2:**
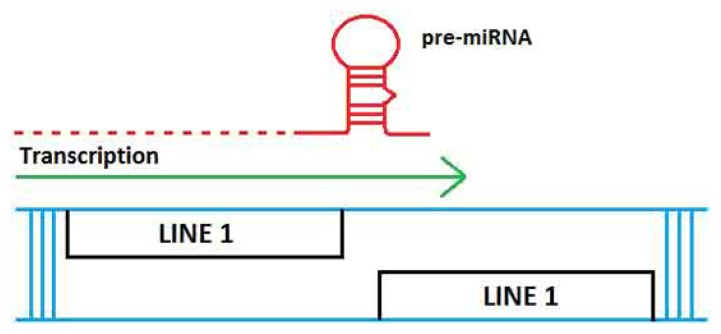
Schematic of proposed origin of TE-based miRNAs. When two related but not identical LINE1elements insert themselves near each other, but on opposite strands of the DNA (in blue), they can create a precursor miRNA containing an imperfect stem-loop upon transcription (red line). The pre-miRNA is shown above the arrow and transcription is indicated from the positive strand LINE1. The stem is potentially recognized and processed by the endogenous RNAi machinery. The pre-miRNA stem-loop depicted is representational. Modified from [26].

A more recent study involved a comprehensive analysis of the genomic events responsible for the formation of ~15,000 annotated miRNAs against the principle datasets for TEs and ncRNAs and found 2392 (~15%) TE-based miRNAs [26]. The majority of these TE-based miRNAs may have originated via the proposed mechanism depicted in Figure 2.

The authors further investigated the exact TE origins of these 2392 miRNAs and showed that DNA transposons comprise the TE most frequently responsible for miRNA generation (891); others were: LTR Retrotransposon (414), Non-LTR Retrotransposon (814), LINE (312), SINE (353), Satellite (137) and others (136). This last category (“Other”) had significant sequence identity to known noncoding RNA sequences (e.g., snoRNAs, scaRNAs, tRNAs). Lastly, a hypothetical scheme proposes that the regulatory miRNAs may have arisen via selective subfunctionalization created by the associated benefit of regulating host genes containing portions of TEs.

Using the miRBASE database, a more recent study sought to map all miRNA precursors to several genomes and to determine the repetition and dispersion of the corresponding loci, as well as the repetitive elements overlapping these loci. To facilitate this analysis an automatic method called ncRNAclassifier was used in order to classify the relationship of TEs with pre-ncRNAs [27]. By applying this method, a correlation between the number of pre-ncRNA candidates and the presence of TEs was determined using six genomes (frog, human, mouse, nematode, rat and sea squirt). The results indicate that 235 and 68 mis-annotated pre-miRNAs correspond completely to TEs out of 1426 human and 721 mouse pre-miRNAs of miRBase (10.0 release), respectively. Further, the various types of TEs involved were also identified and include (MADE1 and other MITEs, DNA transposons, LTR/ERV, CR1/RTE, L1, SINE, other non-LTR). Lastly, the authors suggest that the ncRNAclassifier can be openly used to determine if a given ncRNA hairpin sequence corresponded to a TE sequence.

An investigation of the TE origins of miRNAs focused on the MER (MEdium Reiteration frequency), interspersed repeats in the genomes of primates, rodentia, and lagomorpha) transposon-derived miRNAs in human genome. Once again, a bioinformatics approach was undertaken to identify the specific miRNAs that are derived from palindromic MERs, by analyzing MER paralogs in human genome. Results from this study identified three miRNAs derived from MER96 located on chromosome 3, and MER91C paralogs located on chromosome 8 and chromosome 17 [28]. More importantly, this study also experimentally validated the interactions between these MER-derived miRNAs with AGO1, AGO2, and AGO3 proteins (involved in gene silencing and act as the catalytic component of the RNA induced silencing complex [RISC]).

Lastly, there are additional classes of small ncRNAs that originated from TEs and/or consist of TE sequences, e.g., certain piRNAs and the specialized SINE and Alu transcripts that function as small ncRNAs; these are discussed below with respect to their functional roles.

In summary, a sizable proportion of miRNAs appear to be derived from TEs. It is highly probably that future bioinformatic analyses will increase the number of miRNA-transposable element relationship as not all miRNAs have been discovered and most likely, all consensus repetitive elements have not yet been described. As ncRNAs serve such a critical regulatory role, TE colonization of the genome has given rise to a number of regulatory processes, several of which we discuss here.

## 3. Interaction of TEs with ncRNAs—Functional and Disease-Related Significance

### 3.1. Alu Element Embedded in Long ncRNAs and mRNAs—Crucial Role in Target mRNA Decay

**Figure 3:**
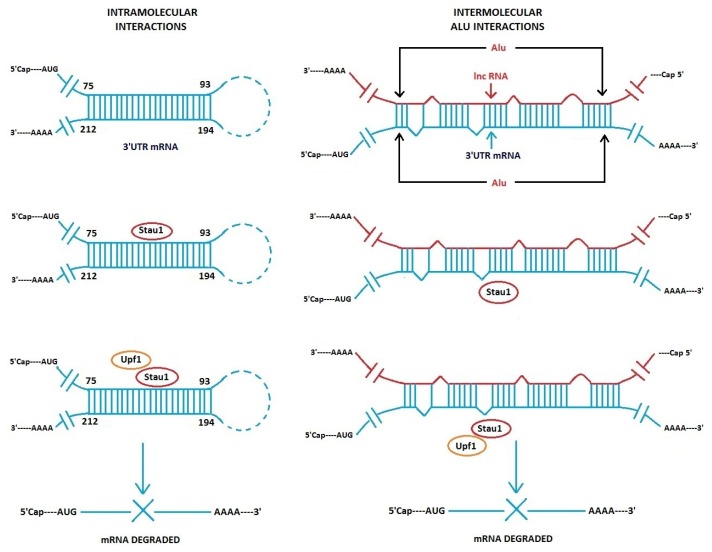
Schematic of binding of Stau1 to 3′ UTR mRNA intramolecular stem (*arf1* mRNA) (**left**) and to intermolecular stem formed by Alu sequence in lncRNA with Alu sequence within 3′UTR mRNA. Upf1is an RNA helicase. The two RNA duplex stems shown are not drawn to scale. Modified from Gong and Maquat [30].

Gong and Maguat revealed the importance of Alu intermolecular base-pairing to lncRNA-induced degradation of mRNA [29,30]. By computational analysis, Alu sequences are found present in ~380 lncRNAs in HeLa cells [30]. In addition, mRNAs were identified that contain an Alu sequence in their 3′UTR regions. Certain mRNAs are targets of the double-stranded RNA binding protein Staufen1 (Stau1), which can induce degradation of the mRNAs. Co-immunoprecipitation experiments show an Alu-containing lncRNA, which originates from chromosome 11, binds to and decreases the abundance of target messages, *i.e.*, plasminogen activator inhibitor type 1 (SERPINE 1) mRNA and an mRNA that encodes an unknown protein termed FLJ21870. Both these mRNAs have an Alu sequence in their 3′UTRs. By secondary structure modeling, it was shown that Alu sequences in lncRNA can base-pair to Alu sequences in the 3′UTR of target mRNAs by intermolecular base-pairing with a stable −Δ G (Figure 3). This pairing is imperfect and contains bulged positions. The intermolecular stem structure, formed by the interaction between the Alu sequences present in the lncRNA and in the 3′ UTR of the mRNA serves as the binding site for Stau1, which subsequently recruits UPF1, a protein required to initiate mRNA decay. Several hundred other lncRNAs contain Alu sequences and have the potential to base-pair with Alu-containing mRNAs, but possible functions of the majority of the several hundred Alu-containing lncRNAs are unknown.

Alu/Alu sequence pairing is not the only interaction seen with Stau1-binding mRNAs, but it represents an important “variation on a theme”. In previous experiments with another Stau1-binding mRNA that contains no Alu sequences but a perfect 19 bp stem in the 3′UTR, it was shown that Stau1 binds the perfect 19 bp stem that is formed intramolecularly between distal sequences in the 3′UTR of the mRNA [31] (Figure 3, left). ARF1 is an ADP-ribosylation factor 1 protein, and the *arf1* mRNA transcript has a Stau1-binding site. The 19 bp stem is phylogenetically conserved in different mammalian species. The question remains, how many Stau1-binding mRNAs are targets for decay via intramolecular bp within a message and how many by intermolecular Alu/Alu pairing. As mentioned, there are several hundred lncRNAs that have Alu sequences. In addition, in a related topic, it should also be pointed out that inverted Alu elements are found in many human mRNA 3′UTR sequences. These can form double-stranded intramolecular stems, and they appear to affect mRNA translation efficiency [32].

Thus, mRNAs that have a Stau1 binding site, whether it is a formed intermolecularly via Alu/Alu lncRNA/mRNA imperfect base-pairing or by perfect Watson–Crick intramolecular pairing within the mRNA can be targeted for degradation. This raises an interesting question concerning the specificity of Stau1 and its recognition sites on duplex RNA stems and imperfect *vs.* perfect double stranded stems. The probability of mistakes in recognition must be very low, yet two or more types of RNA tertiary structures are recognized with great accuracy. Crystal structures of perfect and imperfect double-stranded RNA/Stau1-protein complexes would be of major interest. Gleghorn *et al*. have already determined crystal structures of Stau1 and showed that dimerization of Stau1 occurs by a degenerate dsRNA-binding domain on Stau1 [33]. And in another recent study, it was revealed that dimerization can also involve Stau2 [34].

In another study from this laboratory, rodents appear to use the same mechanism of mRNA regulation involving intermolecular imperfect base-pairing between lncRNAs and mRNAs, only this occurs via SINE elements B1, B2 or B4 found at 3′UTRs and in lncRNAs [35].

### 3.2. Long Non-Coding Antisense RNA Controls mRNA Translation—Importance of Embedded SINE/Alu Repeats

Carrieri *et al.* determined that a long ncRNA transcript, which is partially antisense to ubiquitin carboxy-terminal hydrolase L1 (Uchl1), is essential for increased translation of uch1 mRNA [36]. Uch1 is a neuron associated protein in mammals and may be involved in neurological disease formation [37,38]. Two segments of the antisense lncRNA are crucial for function: the 5′ end of the lncRNA transcript that overlaps the uch1 sense transcript, and SINEB2 and Alu repeat segments located downstream on the antisense lncRNA. By using a bioinformatics approach, 31 antisense lncRNAs have been pinpointed that contain SINE/Alu sequences in their 3′ end half regions. These RNAs can potentially base-pair to sense transcripts via their 5′ ends to the 3′UTR of mRNAs, in a similar manner as uch1 sense RNA/antisense RNA transcript pairing occurs. However the mechanism by which the SINE sequences act to control translation of uch1 mRNA has not been determined, but a hint comes from data showing the orientation of the SINE in the antisense lncRNA is important in rescue experiments [36]. This implies a possible RNA/RNA base-pairing mechanism. This work may define a separate class of regulatory lncRNAs in mammals that appears to differ from the Alu-containing lncRNAs described by Gong and Maquat [29].

### 3.3. Point Mutation in LINE-1/Alu Element Embedded in a lncRNA Results in Lethal Brain Disease

A primate conserved LINE-1 sequence is found embedded in a lncRNA that maps to human chromosome 8p22 [39]. This LINE sequence also overlaps with an Alu sequence. The lncRNA constitutes a unique transcript originating from an intron. This RNA most likely has regulatory functions. A rare single point mutation, A to G in the LINE-1/Alu sequence is associated with brainstem cell atrophy, a genetic abnormality that results in lethal infantile encephalopathy in humans [39]. The LINE-1 is a degenerate retrotransposon and assumed not to be mobile. It was experimentally determined that in patient brain tissues, the expression of the mutant lncRNA was reduced nearly 10-fold relative to unmutated RNA control levels. mRNAs of two genes that map in the same locus as the lnRNA were found to be unchanged. In addition, knockdown experiments against wild-type lncRNA using siRNA showed a significant increase in apoptotic cells.

Several hypotheses have been presented to explain the drastic phenotypic effects of the single base-pair change in the LINE-1/Alu sequence [39]. One is that piRNAs accidentally target the lncRNA transcript via base-pairing with the mutated LINE-1/Alu sequence and induce silencing of the lncRNA. Another involves inadvertent SRP protein recognition of the mutated sequence, which resides in a conserved internal loop of the Alu secondary structure embedded in the lncRNA. This loop is also present in the 7SL RNA secondary structure.

Thus, this is an example of a point mutation in an embedded TE in a lncRNA sequence that produces human disease. What is not known is what exact role the unique lncRNA plays in normal cell functions and the normal function of the embedded LINE-1/Alu sequence, although there may be involvement in regulatory networks during brain development [39].

### 3.4. Facioscapulohumeral Muscular Dystrophy (FSHD)-Involvement of Tandem Repeats and Long ncRNA

Repeat elements in primate genomes are primarily of two types: repeats of transposable element integration into the genome and tandem repeats (microsatellites, minisatellites and macrosatellites). There is evidence that ~25 percent of minisatellite tandem repeats are derived from TEs [18]. The evolutionary origin of most macrosatellites is uncertain, however the macrosatellite tandem repeat at human chromosome region 4q35 discussed below has an interesting origin and possibly related to a retrotransposition. It represents a remnant of an unprocessed mRNA from a primate ancestral retrogene. The retrogene was lost but the retrotranscribed unprocessed mRNA (which included introns) was retained. It proceeded through an expansion in the last 25 million years and was greatly multi-copied in Homo sapiens [40–43].

The tandem repeat macrosatellite sequences at chromosome region 4q35 and a chromatin associated long ncRNA are intimately involved in a human genetic disorder termed facioscapulohumeral muscular dystrophy (FSHD) [17,44]. This is a process perhaps best described as a “cause and effect” mechanism involving loss of repeat sequences, the activation of a lncRNA and resultant epigenetic changes in FAHD patients.

**Figure 4:**
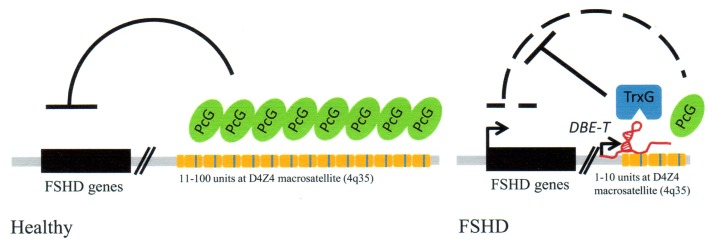
Involvement of repeat units (in orange) and lncRNA (in red) in FSHD muscular dystrophy. DBE-T, lncRNA; TRG, Trithorax proteins; other terms are defined in text. From Casa and Gabellini [17]. Reprinted with permission of publisher.

Normal individuals carry a certain number of repeats (11–100) in the facioscpulohumeral (FSHD) locus. FSHD patients have a decreased number of repeats [17]. The FSHD locus maps to the chromosomal region 4q35. The region containing the repeat sequences is termed D4Z4 (Figure 4). A lncRNA termed DBE-T is partly encoded by the D4Z4 repeat locus at its 3′ end. The genomic repeat sequences serve as binding sites for Polycomb proteins (PcG). PcG proteins have multiple functions but are epigenetic suppressors and are needed to suppress the *FSHD* genes that are silent in normal individuals. However, when there is a loss of genomic repeats resulting in less than 11 repeats, as is found in FSHD patients, DBE-T lncRNA transcription is activated. The RNA is normally silent and is only detected in FSHD patients. DBE-T lncRNA recruits Ash1L, a histone methytransferase to the FHDS locus, which subsequently results in transcription of locus genes due to epigenetic changes in chromatin. Thus these are changes involving the loss of repeat sequences in the D4Z4 region, transcription of a lncRNA with resultant chromatin remodeling and transcription of normally silent genes from the FSHD locus (Figure 4).

This fascinating study raises questions regarding the cell’s reliance of repeat sequences for crucial epigenetic regulation. The D4Z4 region is highly variable in normal individuals, again, 11–100 copies of repeats. Genetic rearrangements appear to have take place in the 4q35 region in FSHD patients [45]. It seems that the cell’s reliance on variable number of repeat sequences, as in the D4Z4 region for regulation appears to be a flawed mechanism used for epigenetic silencing. There are also other genetic abnormalities involving deletions in unstable chromosomal regions that have repeat sequences, as in chromosome locus 22q11.2 in DiGeorge Syndrome [46]. Although these repeat regions widely differ in structure and properties, e.g., DiGeorge syndrome can involve rearrangements between palindromic AT-rich repeats, it appears that genomic repeat sequences may constitute weak points in terms of maintenance of genomic fidelity, even though they have their important functions in the cell.

### 3.5. Embedded Alu Sequences Can Take Part in Alternative Splicing and A to I Editing in Human mRNAs

As opposed to other embedded Alu sequences described in this manuscript, the association of Alus with alternate splicing and RNA editing represent nuclear processes that Alu sequences participate in. Alu sequences are found in exons in about 5% of alternatively spliced mRNAs [47]. The presence of an Alu in exons of pre-messenger RNA transcripts can provide alternative splice sites and parts of the embedded Alu sequences can be incorporated into the processed mRNA [48–51].

Approximately 45 percent of Alu sequences are found in introns in both 5′ to 3′ and reverse orientations and are present in multiple copies [47,52]. These Alu sequences can potentially form double-stranded stems within a transcript when two Alu RNAs are in antiparallel orientation [53]. This enables RNA editing to take place [54] and can lead to premature stop codons or changes in codon reading [47]. Alus are prominent targets for RNA editing [54]. This is an example of how Alu RNA secondary structure can participate in altering molecular processes.

### 3.6. Human Endogenous Retrovirus (HERV) LTR Transcripts

About 8% of the human genome consists of human endogenous human retroviruses (HERV) [55]. HERVs cannot produce a viable virus due to mutations, but its associated LTR transposons serve a vital role in cell transcription. In addition, the human genome contains several thousand copies of single long terminal repeats (sLTRs), which originally stem from HERV [55]. These sLTRs carry no viral genes but can function as promoters and enhancers when found upstream of genes. However some sLTRs are situated in introns and are transcribed into RNA. Xu and co-workers, while studying the expression of HERV-9 U3 sLTR show that sLTR RNA transcripts are both sense and antisense RNAs, but the U3 sLTR antisense transcript can bind key transcription factors involved in cell proliferation. The sense sLTR RNA does not bind transcription factors. Importantly, malignant cells express lower levels of antisense sLTR RNA relative to sense transcripts than normal cells. The antisense sLTR RNA, which is ~550 nt appears to be a novel sLTR RNA species. The authors propose that the antisense sLTR lncRNA serves as a trap for some cell proliferation transcription factors. This may have significance in terms of a possible lack of inhibition of growth in cancer cells. Thus this is an example of a regulatory lncRNA that is encoded by a transposon (sLTR), but binds to and inactivates proteins and not other RNAs. Importantly, this ncRNA may play a crucial role in cell proliferation [55].

### 3.7. Interrelatedness between HERV LTRs and Intergenic Long Non-Coding RNAs

In a different study concerning HERV LTRs, other TEs and lncRNAs, Kelly and Rinn provide a comprehensive analysis of human TE sequences in long intergenic non-coding RNAs (lincRNAs) and conversely, the presence of lincRNAs sequences in transposons [56]. About 7700 lincRNAs overlap TEs and about 1530 lincRNAs are devoid of TEs; thus about 80 percent of human lincRNAs are associated with TEs. lincRNAs display a strikingly non-random association with transposable elements; the majority overlap human endogenous retrovirus (HERV) LTRs and a small minority are associated with LINE or SINE elements.

Interesting observations were made on the orientation of HERV transposons relative to lincRNAs and expression in specific cells. A large number of HERV LTRs are situated at the transcriptional start sites (TSS) of lincRNAs and in the sense orientation. This suggests that HERV LTRs provide regulatory signals for lincRNAs. lincRNAs display a marked stem cell specificity in expression, but lincRNAs that have no LTR associations are expressed highest in testes. On the other hand, lincRNAs that contain Alu sequences are expressed in all cell lines but testes. lincRNAs most likely function in specific tissues, but Alu-containing lincRNAs may be deleterious in testes, as they are not expressed in these tissues. Thus, there is a tissue-specificity in expression [56]. TEs may work “hand-in-hand” with lincRNAs as functional units in particular cells.

### 3.8. Regulatory Non-Coding Circular RNAs

Circular RNAs were first characterized in human and other mammalian cells about 20 years ago [57,58], however they were initially detected in electron micrographs over 30 years ago [59]. These RNAs consist of scrambled protein coding exons, *i.e.*, the order of exons is not the same as in the genomic sequence of protein coding regions. Scrambled exon sequences were discovered in RNA transcripts in rodents and humans [60]. Subsequently, additional cirRNAs were found [61–63]. Recently, by using deep sequencing of RNA techniques and a bioinformatics approach, Saltzman *et al.* [64] discovered several hundred circular RNAs in human cells and surprisingly, Jeck *et al.* [65] using circular enrichment techniques as well as bioinformatics determined that greater than 14% of human fibroblast gene transcripts are cirRNAs (over 25,000 circular transcripts).

Although circular RNAs arise from protein-coding regions, they do not encode proteins. They are thus a separate class of long non-coding RNAs. Functions of cirRNAs were not elucidated for over 20 years since their discovery. However, the field has now moved dramatically, with two laboratories determining that some circular RNAs serve as “sponges” that can bind approximately 70 microRNAs and thus inactivate the microRNAs [6,7]. This shows that circular RNAs have regulatory functions, *i.e.*, they “regulate the regulator”, the microRNAs.

Via bioinformatics analyses, it was shown that Alu elements are found in upstream and downstream introns that straddle the exons that are circularized, and that Alu sequences tended to be inverted and thus complementary [65]. Intron pairing may contribute and be essentially to circularization of exons by complementary base-pairing between Alu elements in the upstream and downstream introns. If this is so, then Alu elements play a major role in formation of circularized RNAs. Related to this, there is precedent for Alu pairing in intons during alternative splicing [53].

### 3.9. piRNAs—Known Regulators of TEs

piRNAs are a class of small non-coding RNAs that are 26–31 nt. They interact with Piwi proteins, hence their name. The Piwi family is regulatory proteins that were originally defined in Drosophila as P-element induced wimpy testis [66]. piRNAs are abundantly found in germ line cells, especially in mammals, e.g., several million piRNAs are found in mammalian testes. Genetic regions that encode piRNAs consist of clusters. These clusters have repeats of piRNA sequences and there can be as many as 1000 copies of piRNAs in a cluster. piRNAs are processed from long precursors transcripts but little is known of the biogenesis of piRNAs and the number and functions of the associated proteins.

Some piRNA clusters consist of transposon or remnants of transposon sequences. Thus piRNAs can have sequences complementary to transposon sequences and can recognize their targets by base-pairing, either by perfect or imperfect base-pairing. A major role of the piRNA/Piwi protein complex in germ line cells is to protect cells from invading transposons. This is a type of “genetic immune system” that is found in both eukaryotes and prokaryotes. For example, the CRISPR complex in bacteria and archae functions by a comparable mechanism (albeit with significant variations on a theme) to protect cells from invasion by plasmids and viruses [67–69]. Both the piRNA/Piwi and CRISPR immune complexes function by an RNA-based mechanism.

piRNA functions have been studied in detail in *Drosophila*, *C. elegans* and mammalian cells. Functions are complex and may differ in different species, but a large fraction of piRNAs represent antisense transcripts to transposon transcripts [70–72]. A basic mechanism of action of piRNAs, first deduced in *Drosophila*, has the following scenario: when the cell is previously exposed to a TE but now experiences an overload of this transposon, piRNAs containing complementary sequences to the TE will base pair with and induce degradation of the TE RNA via the Piwi proteins. When the cell encounters a transposon that it has not been exposed to before, the TE by chance, may incorporate into the DNA in a piRNA-encoding cluster and thus its sequence can become part of the piRNA cluster (however, we are not aware that the probability of incorporation has been experimentally determined). Via the same mechanism mentioned above, piRNA transcripts that are antisense to the new transposon RNA induce degradation of the TE RNA via the piRNA–Piwi complex [71]. Thus, this is an immune system that helps keep transposons in check [70]. It is of interest that cell survival depends in part, on the probability of incorporation of the TE into a piRNA cluster, *vs.* the probability of insertion into and inactivation of an essential gene. However, other protective mechanisms also operate to limit TE activity.

Additional processes of piRNAs have been determined. In nematodes, piRNAs detect a TE sequence via imperfect base-pairing and then induce another small RNA class, termed 22G-RNAs to silence a transposon [73]. Some processes involve epigenetic mechanisms. For example, in Drosophila, nuclear piRNAs can target a transposon and thus direct Piwi proteins to repression chromatin and thus transcription of the TE [74]. Additionally, piRNAs may also induce the methylation of TE LINE-1 DNA in humans. This can prevent transcription of the transposon and thus assure that the TE DNA will remain dormant and not be expressed [75].

The piRNA/Piwi complex is also essential in genetic imprinting in the case involving DNA methylation of the imprinted locus Ras protein-specific guanine nucleotide-releasing factor 1 (Rasgfr1) locus in mouse germ line cells [76]. Mutants affecting piRNA expression correlate with defects in DNA methylation of Rasgrf1. The differentially methylated region (DMR) associated with Rasgfr1 contains a LINE1 retrotransposon and sequences consisting of 23–31 nt small RNAs; these correspond to piRNAs. Yet, a different locus on chromosome 7 also has a region that produces piRNAs that have a good match to the Rasgfr1 DMR piRNA sequences. The authors propose that piRNAs generated from chromosome 7 target the retrotransposon in the DMR of the imprinted Rasgrf1 locus and that chromosome 7 piRNAs may direct methylation of the Rasgrf1 locus [76]. Thus piRNA, in addition to being a post-transcriptional regulator may also be involved in epigenetic regulation of chromosomal genes and genetic imprinting.

### 3.10. SINE/Alu Transcripts Function as ncRNAs in Gene Regulation at the Transcription Level

The non-autonomous retrotransposon SINE sequences are transcribed into small ncRNAs, but like some piRNAs, they can function at the level of transcription. A SINE transcript termed B2, which is found in the nucleus of mice was shown to be 177 nt. This B2 RNA transcript is conserved in rodents. This RNA binds polymerase II during the heat shock response, disrupts the polymerase/promotor interaction and represses transcription from protein gene promoters [77–79]. It is unclear whether the ncRNAs also interact with the promoter sequence. The human counter part, an Alu RNA, functions in a similar manner during heat shock, even though it’s nucleotide sequence and secondary structure differs from B2 RNA [47,77]. Expression of both these small ncRNAs is increased during the heat shock response.

In other studies, a processed human Alu RNA has a sequence that is identical to that of a piRNA present in mammalian testes [80]. The processed transcript, termed piAluRNA is found in the nucleus of human adult stem cells, appears to interact with several nuclear proteins and may be involved in several processes. These include transcription, chromatin organization, organelle organization, DNA repair and cell cycle control [80]. An RNA affinity assay with synthetic oligonucleotides representing a segment of the piAluRNA and high-resolution mass spectrometry-LC-MS were used to identify interacting proteins. Functional studies are needed, but the current binding data strongly suggest the involvement of piAluRNA in several of these nuclear functions. These studies may greatly extend the roles of small ncRNAs in cells.

It is important to point out that binding and repression of proteins by ncRNAs also occurs in prokaryotes. For example, the bacterial ncRNA 6S RNA regulates RNA polymerase by binding sigma factor 70 factor and subsequently repressing RNA polymerase activity from sigma 70 promoters [81–83]. Thus, this is another example of the basic principles of molecular regulation that encompass all biological kingdoms.

## 4. Conclusions

We presented examples of ncRNAs originating from TEs, such as miRNAs derived from MADE1 TE. In addition, there are piRNAs that consist of TE sequences and processed SINE and/or Alu transcripts that function as small ncRNAs. Some findings show TE-derived miRNAs to be less conserved than non-TE-derived miRNAs [21], which may imply a species-specific function of TE-derived ncRNAs. There is growing evidence for the meshing of TE sequences with ncRNAs involving both structure and function, and this association has resulted in formation of new regulatory pathways. It is obvious that TE transcripts are of an enormous asset to organisms, either as embedded sequences in ncRNAs or as individual RNAs. The interaction between TEs and ncRNAs could be looked at as a “symbiotic partnership” between the cell and transposable elements involving structure and function.

Cells offer TEs the means to multiple and maintain stability. Nevertheless, when active, TEs become overabundant and can become a threat to survival. The cell then elicits mechanisms to limit their replication. In this process, cells often use the TE sequences themselves to limit proliferation, as in the case with piRNAs involved in the genetic immune system [71].

Of special significance is that ~80% of long intergenic non-coding RNAs are associated with TEs, and in a nonrandom fashion [56]. They most likely serve functional roles, e.g., retrotransposon LTRs may provide regulatory signals for associated lincRNAs. This may be the tip of the iceberg in terms of TE/lincRNA functions as there are thousands of transcripts found in humans.

Most known ncRNA/target RNA interactions consist of short imperfect base-paired stems, and many miRNAs can bind to and regulate multiple target sequences. But this raises the question of the probability of making mistakes and targeting the wrong RNA. Other factors such as RNA-binding proteins also contribute to RNA recognition and stable formation, but the probability of mistakes must be very low, as imperfect RNA/RNA interactions appear to be highly specific. For example, the Alu-ncRNA/Alu-target RNA recognition must be as stable and specific as that of the intramolecular Watson–Crick base-paired stem recognized by Stau-1 in mRNA-induced degradation in human cells [29,30]. These stems are an interesting example of divergent RNA/RNA structures that bind the same RNA-binding protein. Three-dimensional RNP structures are needed to understand duplex conformations and protein binding sites on the two types of RNA stems.

Multiple ncRNA/target RNA pairings by the same ncRNA are also found in prokaryotic interactions with binding of ncRNAs to different target sequences and with different predicted base-pairings, e.g., see [84,85]. Thus stable short ncRNA/target RNA sequence pairings and multiple targeting are found in prokaryotes and eukaryotes. There is a beauty in the specificity and stability of small imperfect RNA/RNA pairings, and with employment of transposable elements in this binding process, and at least in eukaryotes, the cell appears to also use TE sequences to a significant extent in this form of binding.

It is of interest that some TE ncRNAs have been shown to bind proteins. For example, the lncRNA from an LTR retrotransposon situated in an intron binds transcription factors involved in cell proliferation [55]. The B2 ncRNA from an Alu transcript binds polymerase II [47]. The piAlu RNA binds nuclear proteins [80]. Thus transposon RNA transcripts show versatility in function in that these can also repress nuclear protein functions. The 6S RNA in bacteria, although not a TE transcript, is an example of a prokaryotic ncRNA that binds and inhibits the bacterial polymerase enzyme. This adds to the universality of ncRNA-related regulatory mechanisms in biological species. The 6S RNA was the first ncRNA to be sequenced [86], albeit its function was not determined until ~30 years later [81]!

The LINE-1/Alu element in a human lncRNA plays a pivotal role in formation of disease. A single mutation in the embedded TE causes human brainstem atrophy [39]. Whether the embedded LINE-1/Alu element is more prone to mutation than the rest of the lncRNA is not known, but this shows that a point mutation in a TE is the cause of atrophy and death and not a mutation in a protein gene. This adds to the spectrum of mutations that cause human disease, *i.e.*, non-protein-coding genomic sequences can be important factors in human disease. Related to this, the deregulation of lncRNA transcription in diseases such as cancer has recently been highlighted [87,88]. As there are thousands of ncRNAs associated with TEs whose functions have not been determined, the future may possibly hold some interesting surprises with respect to diseases that may have an aberrant ncRNA etiology.

## References

[1] Mattick J.S. (2012). Rocking the foundations of molecular genetics. Proc. Natl. Acad. Sci. USA.

[2] Djebali S., Davis C.A., Merkel A., Dobin A., Lassmann T., Mortazavi A., Tanzer A., Lagarde J., Lin W., Schlesinger F. (2012). Landscape of transcription in human cells. Nature.

[3] Wang Z., Gerstein M., Snyder M. (2009). RNA-Seq: A revolutionary tool for transcriptomics. Nat. Rev. Genet.

[4] Habegger L., Sboner A., Gianoulis T.A., Rozowsky J., Agarwal A., Snyder M., Gerstein M. (2011). RSEQtools: A modular framework to analyze RNA-Seq data using compact, anonymized data summaries. Bioinformatics.

[5] Derrien T., Johnson R., Bussotti G., Tanzer A., Djebali S., Tilgner H., Guernec G., Martin D., Merkel A., Knowles D.G. (2012). The GENCODE v7 catalog of human long noncoding RNAs: Analysis of their gene structure, evolution, and expression. Genome Res.

[6] Memczak S., Jens M., Elefsinioti A., Torti F., Krueger J., Rybak A., Maier L., Mackowiak S.D., Gregersen L.H., Munschauer M.N. (2013). Circular RNAs are a large class of animal RNAs with regulatory potency. Nature.

[7] Hansen T.B., Jensen T.I., Clausen B.H., Bramsen J.B., Finsen B., Damgaard C.K., Kjems J. (2013). Natural RNA circles function as efficient microRNA sponges. Nature.

[8] Di Leva G., Garofalo M., Siregar Y. (2013). Non-Coding RNAs and Cancer. Oncogene and Cancer—From Bench to Clinic.

[9] Mizuno T., Chou M.Y., Inouye M. (1984). A unique mechanism regulating gene expression: Translational inhibition by a complementary RNA transcript (micRNA). Proc. Natl. Acad. Sci. USA.

[10] Andersen J., Forst S.A., Zhao K., Inouye M., Delihas N. (1989). The function of micF RNA: micF RNA is a major factor in the thermal regulation of OmpF protein in *Escherichia coli*. J. Biol. Chem.

[11] Schmidt M., Zheng P., Delihas N. (1995). Secondary structures of *Escherichia coli* antisense *micF* RNA, the 5′-end of the target ompF mRNA, and the RNA/RNA Duplex. Biochemistry.

[12] Cowley M., Oakey R.J. (2013). Transposable elements re-wire and fine-tune the transcriptome. PLoS Genet.

[13] Goodier J.L., Kazazian H.H. (2008). Retrotransposons revisited: The restraint and rehabilitation of parasites. Cell.

[14] Lander E.S., Linton L.M., Birren B., Nusbaum C., Zody M.C., Baldwin J., Devon K., Dewar K., Doyle M., FitzHugh W. (2001). Initial sequencing and analysis of the human genome. Nature.

[15] Doudna J.A. The Doudna Lab. Translational control by mRNA secondary structure—Alu-element regulated miRNA interactions.

[16] De Koning A.P., Gu W., Castoe T.A., Batzer M.A., Pollock D.D. (2011). Repetitive elements may comprise over two-thirds of the human genome. PLoS Genet.

[17] Casa V., Gabellini D. (2012). A repetitive elements perspective in Polycomb epigenetics. Front. Genet.

[18] Ahmed M., Liang P. (2012). Transposable elements are a significant contributor to tandem repeats in the human genome. Comp. Funct. Genomics.

[19] Smalheiser N.R., Torvik V.I. (2005). Mammalian microRNAs derived from genomic repeats. Trends Genet.

[20] Piriyapongsa J., Jordan I.K. (2007). A family of human microRNA genes from miniature inverted-repeat transposable elements. PLoS One.

[21] Piriyapongsa J., Mariño-Ramírez L., Jordan I.K. (2007). Origin and evolution of human microRNAs from transposable elements. Genetics.

[22] Devor E.J., Peek A.S., Lanier W., Samollow P.B. (2009). Marsupial-specific microRNAs evolved from marsupial-specific TEs. Gene.

[23] Yuan Z., Sun X., Jiang D., Ding Y., Lu Z., Gong L., Liu H., Xie J. (2010). Origin and evolution of a placental-specific microRNA family in the human genome. BMC Evol. Biol.

[24] Kapitonov V.V., Jurka J. (1998). MER53, a non-autonomous DNA transposon associated with a variety of functionally related defense genes in the human genome. DNA Seq.

[25] Yuan Z., Sun X., Liu H., Xie J. (2011). MicroRNA genes derived from repetitive elements and expanded by segmental duplication events in mammalian genomes. PLoS One.

[26] Borchert G.M., Holton N.W., Williams J.D., Hernan W.L., Bishop I.P., Dembosky J.A., Elste J.E., Gregoire N.S., Kim J.A., Koehler W.W. (2011). Comprehensive analysis of microRNA genomic loci identifies pervasive repetitive-element origins. Mob. Genet. Element.

[27] Tempel S., Pollet N., Tahi F. (2012). ncRNAclassifier: A tool for detection and classification of transposable element sequences in RNA hairpins. BMC Bioinforma.

[28] Ahn K., Gim J.A., Ha H.S., Han K., Kim H.S. (2013). The novel MER transposon-derived miRNAs in human genome. Gene.

[29] Gong C., Maquat L.E. (2011). lncRNAs transactivate STAU1-mediated mRNA decay by duplexing with 3′ UTRs via Alu elements. Nature.

[30] Gong C., Maquat L.E. (2011). “Alu” strious long ncRNAs and their role in shortening mRNA half-lives”. Cell Cycle.

[31] Kim Y.K., Furic L., Parisien M., Major F., DesGroseiller L., Maquat L.E. (2007). Staufen1 regulates diverse classes of mammalian transcripts. EMBO J.

[32] Capshew C.R., Dusenbury K.L., Hundley H.A. (2012). Inverted Alu dsRNA structures do not affect localization but can alter translation efficiency of human mRNAs independent of RNA editing. Nucleic Acids Res.

[33] Gleghorn M.L., Gong C., Kielkopf C.L., Maquat L.E. (2013). Staufen1 dimerizes through a conserved motif and a degenerate dsRNA-binding domain to promote mRNA decay. Nat. Struct. Mol. Biol.

[34] Park E., Gleghorn M.L., Maquat L.E. (2013). Staufen2 functions in Staufen1-mediated mRNA decay by binding to itself and its paralog and promoting UPF1 helicase but not ATPase activity. Proc. Natl. Acad. Sci. USA.

[35] Wang J., Gong C., Maquat L.E. (2013). Control of myogenesis by rodent SINE-containing lncRNAs. Genes Dev.

[36] Carrieri C., Cimatti L., Biagioli M., Beugnet A., Zucchelli S., Fedele S., Pesce E., Ferrer I., Collavin L., Santoro C. (2012). Long non-coding antisense RNA controls Uchl1 translation through an embedded SINEB2 repeat. Nature.

[37] Choi J., Levey A.I., Weintraub S.T., Rees H.D., Gearing M., Chin L.S., Li L. (2004). Oxidative modifications and down-regulation of ubiquitin carboxyl-terminal hydrolase L1 associated with idiopathic Parkinson’s and Alzheimer’s diseases. J. Biol. Chem.

[38] Barrachina M., Castaño E., Dalfó E., Maes T., Buesa C., Ferrer I. (2006). Reduced ubiquitin C-terminal hydrolase-1 expression levels in dementia with Lewy bodies. Neurobiol. Dis.

[39] Cartault F., Munier P., Benko E., Desguerre I., Hanein S., Boddaert N., Bandiera S., Vellayoudom J., Krejbich-Trotot P., Bintner M. (2012). Mutation in a primate-conserved retrotransposon reveals a noncoding RNA as a mediator of infantile encephalopathy. Proc. Natl. Acad. Sci. USA.

[40] Hewitt J.E., Lyle R., Clark L.N., Valleley E.M., Wright T.J., Wijmenga C., van Deutekom J.C., Francis F., Sharpe P.T., Hofker M. (1994). Analysis of the tandem repeat locus D4Z4 associated with facioscapulohumeral muscular dystrophy. Hum. Mol. Genet.

[41] Clapp J., Mitchell L.M., Bolland D.J., Fantes J., Corcoran A.E., Scotting P.J., Armour J.A., Hewitt J.E. (2007). Evolutionary conservation of a coding function for D4Z4, the tandem DNA repeat mutated in facioscapulohumeral muscular dystrophy. Am. J. Hum. Genet.

[42] Snider L., Asawachaicharn A., Tyler A.E., Geng L.N., Petek L.M., Maves L., Miller D.G., Lemmers R.J., Winokur S.T., Tawil R. (2009). RNAtranscripts, miRNA-sizedfragments and proteinsproduced from D4Z4units: New candidates for the pathophysiology of facioscapulohumeral dystrophy. Hum. Mol. Genet.

[43] Cabianca D.S., Gabellini D. (2010). FSHD: Copy number variations on the theme of muscular dystrophy. J. Cell Biol.

[44] Cabianca D.S., Casa V., Bodega B., Xynos A., Ginelli E., Tanaka Y., Gabellini D. (2012). A long ncRNA links copy number variation to a polycomb/trithorax epigenetic switch in FSHD muscular dystrophy. Cell.

[45] Wijmenga C., Hewitt J.E., Sandkuijl L.A., Clark L.N., Wright T.J., Dauwerse H.G., Gruter A.M., Hofker M.H., Moerer P., Williamson R. (1992). Chromosome 4q DNA rearrangements associated with facioscapulohumeral muscular dystrophy. Nat. Genet.

[46] Emanuel B.S. (2008). Molecular mechanisms and diagnosis of chromosome 22q11.2 rearrangements. Dev. Disabil. Res. Rev.

[47] Ponicsan S.L., Kugel J.F., Goodrich J.A. (2010). Genomic gems: SINE RNAs regulate mRNA production. Curr. Opin. Genet. Dev.

[48] Sorek R., Ast G., Graur D. (2002). Alu-containing exons are alternatively spliced. Genome Res.

[49] Ram O., Schwartz S., Ast G. (2008). Multifactorial interplay controls the splicing profile of Alu-derived exons. Mol. Cell Biol.

[50] Gal-Mark N., Schwartz S., Ast G. (2008). Alternative splicing of Alu exons—Two arms are better than one. Nucleic Acids Res.

[51] Schwartz S., Gal-Mark N., Kfir N., Oren R., Kim E., Ast G. (2009). Alu exonization events reveal features required for precise recognition of exons by the splicing machinery. PLoS Comput. Biol.

[52] Dagan T., Sorek R., Sharon E., Ast G., Graur D. (2004). AluGene: A database of Alu elements incorporated within protein-coding genes. Nucleic Acids Res.

[53] Lev-Maor G., Ram O., Kim E., Sela N., Goren A., Levanon E.Y., Ast G. (2008). Intronic Alus influence alternative splicing. PLoS Genet.

[54] Athanasiadis A., Rich A., Maas S. (2004). Widespread A-to-I RNA editing of alu-containing mRNAs in the human transcriptome. PLoS Biol.

[55] Xu L., Elkahloun A.G., Candotti F., Grajkowski A., Beaucage S.L., Petricoin E.F., Calvert V., Juhl H., Mills F., Mason K. (2013). A novel function of RNAs arising from the long terminal repeat of human endogenous retrovirus 9 in cell cycle arrest. J. Virol.

[56] Kelley D., Rinn J. (2012). Transposable elements reveal a stem cell-specific class of long noncoding RNAs. Genome Biol.

[57] Cocquerelle C., Mascrez B., Hetuin D., Bailleul B. (1993). Mis-splicing yields circular RNA molecules. FASEB J.

[58] Capel B., Swain A., Nicolis S., Hacker A., Walter M., Koopman P., Goodfellow P., Lovell-Badge R. (1993). Circular transcripts of the testis-determining gene Sry in adult mouse testis. Cell.

[59] Hsu M.-T., Coca-Prados M. (1979). Electron microscopic evidence for the circular form of RNA in the cytoplasm of eukaryotic cells. Nature.

[60] Nigro J.M., Cho K.R., Fearon E.R., Kern S.E., Ruppert J.M., Oliner J.D., Kinzler K.W., Vogelstein B. (1991). Scrambled exons. Cell.

[61] Zaphiropoulos P.G. (1996). Circular RNAs from transcripts of the rat cytochrome P450 *2C24* gene: Correlation with exon skipping. Proc. Natl. Acad. Sci. USA.

[62] Li X.-F., Lytton J. (1999). A circularized sodium-calcium exchanger exon 2 transcript. J. Biol. Chem..

[63] Gualandi F., Trabanelli C., Rimessi P., Calzolari E., Toffolatti L., Patarnello T., Kunz G., Muntoni F., Ferlini A. (2003). Multiple exon skipping and RNA circularisation contribute to the severe phenotypic expression of exon 5 dystrophin deletion. J. Med. Genet.

[64] Salzman J., Gawad C., Wang P.L., Lacayo N., Brown P.O. (2012). Circular RNAs are the predominant transcript isoform from hundreds of human genes in diverse cell types. PLoS One.

[65] Jeck W.R., Sorrentino J.A., Wang K., Slevin M.K., Burd C.E., Liu J., Marzluff W.F., Sharpless N.E. (2013). Circular RNAs are abundant, conserved, and associated with ALU repeats. RNA.

[66] Lin H., Spradling A.C. (1997). A novel group of pumilio mutations affects the asymmetric division of germline stem cells in the Drosophila ovary. Development.

[67] Karginov F.V., Hannon G.J. (2010). The CRISPR system: Small RNA-guided defense in bacteria and archaea. Mol. Cell.

[68] Wiedenheft B., van Duijn E., Bultema J.B., Waghmare S.P., Zhou K., Barendregt A., Westphal W., Heck A.J., Boekema E.J., Dickman M.J. (2011). RNA-guided complex from a bacterial immune system enhances target recognition through seed sequence interactions. Proc. Natl. Acad. Sci. USA.

[69] Sashital D.G., Jinek M., Doudna J.A. (2011). An RNA-induced conformational change required for CRISPR RNA cleavage by the endoribonuclease Cse3. Nat. Struct. Mol. Biol.

[70] Brennecke J., Aravin A.A., Stark A., Dus M., Kellis M., Sachidanandam R., Hannon G.J. (2007). Discrete small RNA-generating loci as master regulators of transposon activity in *Drosophila*. Cell.

[71] Malone C.D., Hannon G.J. (2009). Small RNAs as guardians of the genome. Cell.

[72] Simonelig M. (2011). Developmental functions of piRNAs and transposable elements: A *Drosophila* point-of-view. RNA Biol.

[73] Bagijn M.P., Goldstein L.D., Sapetschnig A., Weick E.M., Bouasker S., Lehrbach N.J., Simard M.J., Miska E.A. (2012). Function, targets, and evolution of *Caenorhabditis elegans* piRNAs. Science.

[74] Le Thomas A., Rogers A.K., Webster A., Marinov G.K., Liao S.E., Perkins E.M., Hur J.K., Aravin A.A., Tóth K.F. (2013). Piwi induces piRNA-guided transcriptional silencing and establishment of a repressive chromatin state. Genes Dev.

[75] Sigurdsson M.I., Smith A.V., Bjornsson H.T., Jonsson J.J. (2012). The distribution of a germline methylation marker suggests a regional mechanism of LINE-1 silencing by the piRNA-PIWI system. BMC Genet.

[76] Watanabe T., Tomizawa S., Mitsuya K., Totoki Y., Yamamoto Y., Kuramochi-Miyagawa S., Iida N., Hoki Y., Murphy P.J., Toyoda A. (2011). Role for piRNAs and noncoding RNA in de novo DNA methylation of the imprinted mouse *Rasgrf1* locus. Science.

[77] Espinoza C.A., Goodrich J.A., Kugel J.F. (2007). Characterization of the structure, function, and mechanism of B2 RNA, an ncRNA repressor of RNA polymerase II transcription. RNA.

[78] Yakovchuk P., Goodrich J.A., Kugel J.F. (2009). B2 RNA and Alu RNA repress transcription by disrupting contacts between RNA polymerase II and promoter DNA within assembled complexes. Proc. Natl. Acad. Sci. USA.

[79] Yakovchuk P., Goodrich J.A., Kugel J.F. (2011). B2 RNA represses TFIIH phosphorylation of RNA polymerase II. Transcription.

[80] Blackwell B.J., Lopez M.F., Wang J., Krastins B., Sarracino D., Tollervey J.R., Dobke M., Jordan I.K., Lunyak V.V. (2012). Protein interactions with piALU RNA indicates putative participation of retroRNA in the cell cycle, DNA repair and chromatin assembly. Mob. Genet. Elements.

[81] Wassarman K.M., Storz G. (2000). 6S RNA regulates *E. coli* RNA polymerase activity. Cell.

[82] Wassarman K.M. (2007). 6S RNA: A regulator of transcription. Mol. Microbiol.

[83] Decker K.B., Hinton D.M. (2009). The secret to 6S: Regulating RNA polymerase by ribo-sequestration. Mol. Microbiol.

[84] Holmqvist E., Unoson C., Reimegård J., Wagner E.G. (2012). A mixed double negative feedback loop between the sRNA MicF and the global regulator. Lrp. Mol. Microbiol.

[85] Corcoran C.P., Podkaminski D., Papenfort K., Urban J.H., Hinton J.C., Vogel J. (2012). Superfolder GFP reporters validate diverse new mRNA targets of the classic porin regulator, MicF RNA. Mol. Microbiol.

[86] Brownlee G.G. (1971). Sequence of 6S RNA of *E. coli*. Nat. New Biol.

[87] Hauptman N., Glavač D. (2013). Long non-coding RNA in cancer. Int. J. Mol. Sci.

[88] Gutschner T., Diederichs S. (2012). The Hallmarks of Cancer: A long non-coding RNA point of view. RNA Biol.

